# Severe Late Toxicity After Adjuvant Breast Radiotherapy in a Patient with a Germline Ataxia Telangiectasia Mutated Gene: Future Treatment Decisions

**DOI:** 10.7759/cureus.1458

**Published:** 2017-07-11

**Authors:** Maryam Dosani, Kasmintan A Schrader, Alan Nichol, Sophie Sun, Tamara Shenkier, Zoe Lohn, Gudrun Aubertin, Scott Tyldesley

**Affiliations:** 1 Radiation Oncology, BC Cancer Agency, Vancouver Centre; 2 Hereditary Cancer Program, BC Cancer Agency, Vancouver Centre; 3 Medical Oncology, BC Cancer Agency, Vancouver Centre

**Keywords:** atm, radiotherapy, breast cancer, late effects, ataxia telangiectasia mutated gene, radiation toxicity, late toxicity

## Abstract

Ataxia telangiectasia mutated (ATM) gene mutations may confer increased sensitivity to ionizing radiation and increased risk of late toxicity for cancer patients. We present the case of a 55-year-old female treated with adjuvant breast and regional nodal radiation following lumpectomy and axillary lymph node dissection for stage II invasive ductal carcinoma of the breast. She developed severe telangiectasia, fibrosis, induration, chest wall pain (with evidence of rib fractures on imaging), and painful limitation in her range of motion at the shoulder. She was subsequently found to have a likely pathogenic germline ATM gene mutation. At relapse, she elected to pursue systemic therapy alone for intracranial metastases.

## Introduction

Adjuvant radiotherapy (RT) is an important component of the treatment for breast cancer. While generally well tolerated, some patients develop grade three-four late toxicity to the skin, subcutaneous tissues, chest wall or shoulder/acromioclavicular joints.

Genetic variants may be associated with a susceptibility to late RT toxicity and mutations in the ataxia telangiectasia mutated (ATM) gene constitute a common, but unproven, candidate [1]. The ATM gene is located on chromosome 11q22-23 and belongs to a family of proteins involved in the recognition and repair of double-strand deoxyribonucleic acid (DNA) breaks that can be induced by ionizing radiation, chemotherapy drugs, or oxidative stress [2]. Homozygous or compound heterozygous carriers of mutations in the ATM gene develop ataxia-telangiectasia (AT), an autosomal recessive disorder characterized by cerebellar ataxia, oculomotor apraxia, immunodeficiency, choreoathetosis, conjunctival telangiectasias, sensitivity to radiotherapy, and an increased risk of malignancy. Individuals who are heterozygous for the ATM mutation may have a milder syndrome of increased sensitivity to ionizing radiation [3-4] and increased risk of malignancy [5-6]. The prevalence of ATM mutations is estimated to be 0.5% to 1.0% in Western populations [7].

We present a case of a female who developed severe skin, breast, and chest wall toxicity after adjuvant four-field breast and nodal RT, who was subsequently found to carry a heterozygous ATM mutation. We review the literature on ATM mutations, RT toxicity and risk of breast cancer in patients with ATM mutations and discuss the implications of such findings. Informed consent statement was obtained for this study.

## Case presentation

Initial presentation and treatment

A 55-year-old female presented in 2010 with a palpable right breast mass and right axillary lymph node. She was previously healthy with a family history of colon cancer in her mother (diagnosed at age 55 years) and prostate cancer in her brother (diagnosed at age 63 years). She worked as a gardener.

Biopsies confirmed an invasive ductal carcinoma of the breast. She was treated with right partial mastectomy and axillary lymph node dissection. Her pathological evaluation revealed a 2 cm invasive ductal carcinoma, grade two, that was estrogen receptor positive (3+), progesterone receptor positive (2+), human epidermal growth factor receptor 2 (Her 2) negative (via immunohistochemistry), and positive for lymph vascular invasion. The invasive disease was 2 mm from the closest (posterior) margin. There was also grade two ductal carcinomas in situ reaching 0.1 mm from the posterior margin. Two of nine resected lymph nodes were positive for metastatic disease. The largest was 3 cm with extra-nodal extension. Her disease was pathologically Stage IIA (pT1c pN1a).

Adjuvant treatment included 5-fluorouracil, epirubicin, cyclophosphamide, docetaxel (FEC-D) chemotherapy and adjuvant radiotherapy (RT). Adjuvant radiotherapy (RT) to the breast and regional nodes was prescribed at 45 Gy in 25 fractions to the breast and internal mammary chain nodes and 48 Gy in 25 fractions to the right supraclavicular fossa and axilla. She received an RT boost to the breast tumor bed of 16 Gy in eight fractions. The patient declined endocrine therapy.

Toxicity from radiotherapy

**Figure 1:**
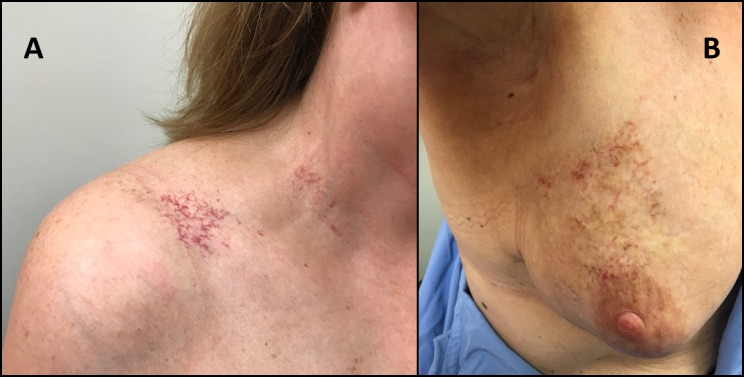
Marked telangiectasias with dense fibrosis following radiotherapy (A) supraclavicular fossa and (B) right breast and axilla

RT was accompanied by a brisk acute skin reaction with moist desquamation around the nipple. Two months following completion of RT, her oncologist noted residual swelling and hyperpigmentation of the breast. The patient reported stabbing pains in the breast, discomfort along the sternum, and decreased range of motion. Four months following completion of RT, she was diagnosed with frozen shoulder (despite participation in physiotherapy), and one year later, had dense fibrosis of the right breast, supraclavicular fossa, and axilla with marked telangiectasias in the neck and upper outer quadrant of the breast (Figure 1). The RT plan was reviewed and it was confirmed that she received the prescribed dose of RT. Surveillance of the breast was done with ultrasounds rather than mammograms because of breast pain and difficulty tolerating compression. She pursued physiotherapy and massage therapy. In 2013, she completed 40 hyperbaric oxygen treatments with significant but transient improvement and surgical lysis of the right chest wall adhesions with some benefit. She continued to have marked fibrosis and telangiectasias around the previous RT area which was accompanied by pain of the right chest wall and breast. The range of motion in her arm remained limited and interfered with her occupational gardening work. A repeat course of hyperbaric oxygen treatment is being considered. 

Relapse and genetic testing

**Figure 2:**
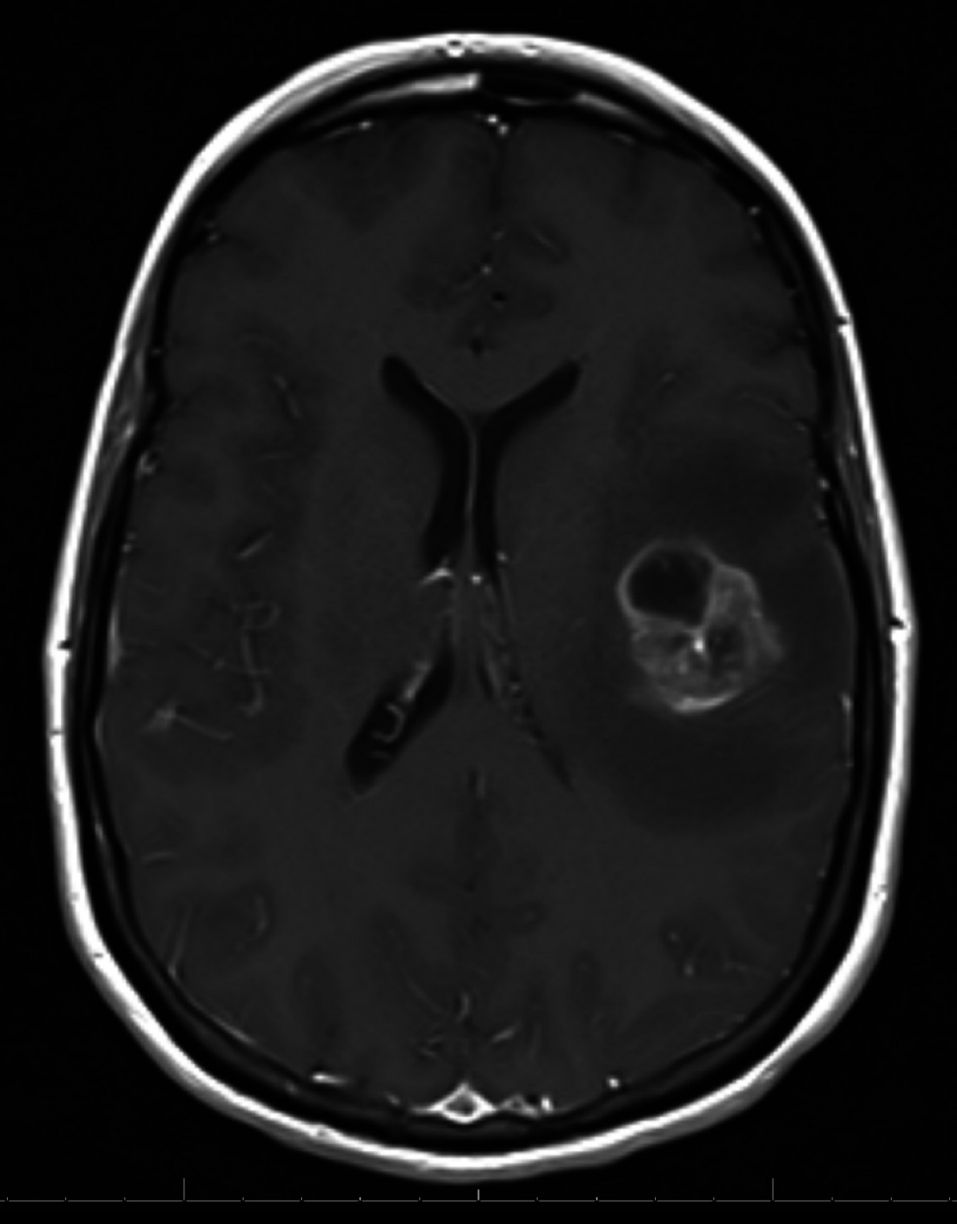
Magnetic resonance imaging (MRI) showing solitary metastasis in the left frontal lobe

Approximately 3.5 years after her initial diagnosis, she developed a solitary 3.2 cm symptomatic brain metastasis that was surgically resected (Figure 2). She declined brain RT citing the significant problems related to her previous RT.

**Figure 3:**
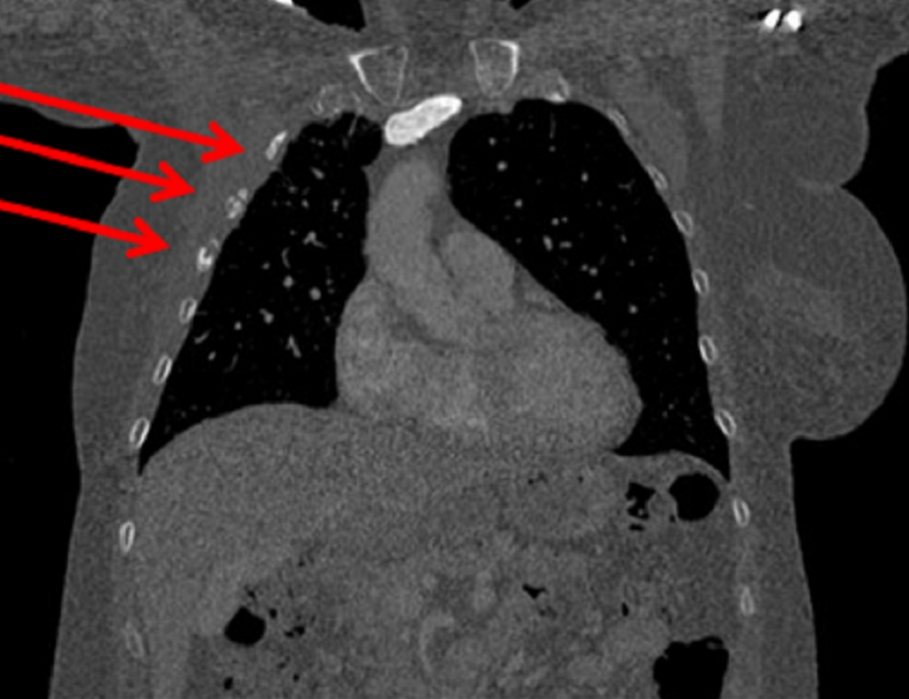
Fractures of the right second to fifth ribs with sclerosis and incomplete bony bridging, attributable to radionecrosis Fractures identified by red arrows

**Figure 4:**
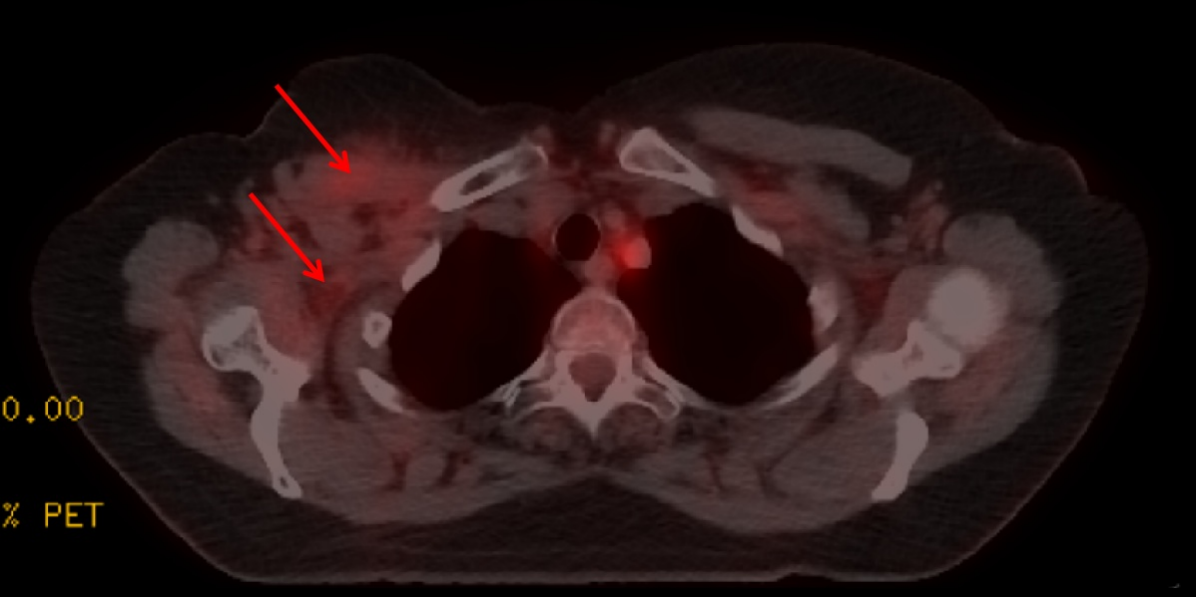
Positron emission tomography (PET) scan showing fluorodeoxyglucose uptake in the right pectoralis major and chest wall consistent with previous radio therapy (RT), but no recurrent disease Arrows indicate regions of fluorodeoxyglucose uptake

Imaging did not show evidence of metastatic disease outside of the brain. Fractures of the right second to fifth ribs with sclerosis and incomplete bony bridging were identified as attributable to radionecrosis (Figure 3). A positron emission tomography-computed tomography (PET) scan showed fluorodeoxyglucose uptake (maximum standard uptake is 2.5) in the right pectoralis major and chest wall, consistent with previous RT (Figure 4). There was no evidence of loco-regional recurrence on computed tomography (CT) or PET.

She took letrozole from January 2014 to August 2016, but this was discontinued due to side effects. Surveillance magnetic resonance imaging (MRI) in October 2016 confirmed an intracranial recurrence at the resection cavity, for which she underwent re-resection. On postoperative MRI, additional dural-based lesions were noted. Postoperative RT was discussed, but she declined because of her previous reaction to the treatment. She elected to start tamoxifen.

Given the severity of her later effects attributed to RT and potential for benefit from further RT, she was referred to the British Columbia (BC) Cancer Agency Hereditary Cancer Program for consideration for ATM genetic testing. This was offered using a 30-gene hereditary cancer gene panel saliva test. Testing confirmed a heterozygous germline likely pathogenic ATM mutation (c.8565_8566delTGinsAA), which was suspected as the causal factor for her RT reaction.

She recently developed the progressive intracranial disease. Whole-brain RT has again been offered, with a discussion around a possible increased risk of side effects because of her previous reaction and ATM mutation. Given her previous toxicity, she has decided against further RT and is currently receiving capecitabine chemotherapy. 

## Discussion

Ionizing radiation causes damage to normal and malignant cells, in part due to DNA double-strand breaks. Non-homologous DNA end-joining, the process responsible for much of the DNA repair process, is impaired in cells with mutated ATM [3]. The risk of increased toxicity from RT for heterozygous carriers of pathogenic ATM mutations is controversial [1,8]. In a retrospective analysis of 46 breast cancer patients treated with adjuvant RT, nine ATM mutations were detected in six patients. All three of the 46 patients with late grade three to four toxicity were found to carry more than one missense variant in the ATM gene. Only one of three patients who were heterozygous for an ATM mutation experienced significant late toxicity [4]. In a German study of 11 breast cancer patients heterozygous for a reported pathogenic ATM mutation, there was no increased toxicity from RT. Of note, this study included one patient treated with brain irradiation for intracranial breast cancer metastases [8]. A limitation of this study relates to more current interpretations of the reported variants that were deemed pathogenic at the time. If tested today, a review of the variants in ClinVar suggests that only two cases would be reported as harboring clearly pathogenic variants [9]. Neither study demonstrated an increase in acute RT toxicity in ATM variant heterozygotes. Differing methodologies, variable expressivity of mutant alleles, and small samples sizes may have hampered our understanding of the question around the risk of RT toxicity attributable to ATM heterozygosity [1].

Impaired repair of DNA double-strand breaks may also confer an elevated risk of cancer in heterozygote carriers of ATM mutations. In a cohort study of UK patients with AT (and carriers), the risk of breast cancer in heterozygote carriers was 2.23 (95% confidence interval [CI], 1.16 to 4.28) compared to the general population, and 4.94 (95% CI 1.90 to 12.9) amongst female under 50 years of age. The relative increased risk of non-breast cancers was 2.05 (95% CI, 1.09 to 3.84) in female carriers and 1.23 (95% CI, 0.76 to 2.00) in male carriers, with some evidence of increased colorectal and stomach cancer risk [5]. In another study of familial breast cancer patients (all negative for the breast cancer gene mutation), the relative risk of breast cancer in ATM mutation heterozygotes was 2.37 (95% CI, 1.51 to 3.78) [6].

For this patient, the heterozygous, likely pathogenic, germline ATM mutation (c.8565_8566delTGinsAA) was identified using a 30-gene hereditary cancer panel testing approach. Such tests are increasingly accessible to the cancer patients and the public due to decreasing costs.

A limitation of this case report is the uncertainty whether our patient’s unusually severe radiation toxicity was attributable to her heterozygous ATM mutation status. Prior studies have more frequently observed severe toxicities in patients with more than one variant or biallelic ATM mutation status. It is possible that her reaction was due to an unrelated type of radiosensitivity. There have been cases of patients diagnosed in adulthood with mild presentations of ataxia telangiectasia (AT)) following severe reactions to adjuvant breast RT [10], raising the small possibility that our patient may have a cryptic ATM mutation in the other ATM gene allele.

Given the uncertainty surrounding the treatment implications for ATM mutation carriers, it is difficult to make clear RT treatment recommendations for these patients. Many patients with heterozygous ATM mutations do not experience increased toxicity from RT. Healthcare professionals should discuss the risks and benefits in the context of the clinical scenario and counsel patients on the potential for increased risk of late toxicity and weigh this against the possible local control or overall survival benefit of RT. Patients and families with germline ATM mutations are at an increased risk of cancer and can benefit from hereditary cancer genetic counseling for individualized screening recommendations.

## Conclusions

Our report describes severe late toxicity following adjuvant breast and nodal radiotherapy (RT) for a patient with stage II invasive breast cancer. While the implications of germline ataxia telangiectasia mutated (ATM) mutation status and susceptibility to RT toxicity are controversial, genetic testing for ATM mutations in patients with previous severe toxicity to RT may be warranted, especially if further RT is being contemplated. With increasing uptake and affordability of hereditary cancer gene panel testing, such findings are likely to become more prevalent. In some cases, testing for ATM mutation status may occur prior to RT. In such cases, patients receiving RT with known heterozygous ATM mutation status may benefit from a further discussion of the benefits and risks of treatment, as well as close observation and early intervention for the late effects of RT if they develop. Index patients and their family members with mutations in this gene should be counseled on an increased risk of future malignancies.
